# ATL I, Acts as a SIRT6 Activator to Alleviate Hepatic Steatosis in Mice via Suppression of NLRP3 Inflammasome Formation

**DOI:** 10.3390/ph15121526

**Published:** 2022-12-08

**Authors:** Danli Kong, Zhenhua Mai, Yongze Chen, Ling Luo, Hao Liu, Le Zhao, Ruixian Huang, Shuang Wang, Rong Chen, Hao Zhou, Hao Chen, Jingjing Zhang, Haibing Yu, Yuanlin Ding

**Affiliations:** 1 Department of Epidemiology and Medical Statistics, School of Public Health, Guangdong Medical University, Dongguan 523808, China; 2 Department of Critical Care Medicine, Affiliated Hospital of Guangdong Medical University, Zhanjiang 524002, China; 3 Department of Gastroenterology, Affiliated Hospital of Guangdong Medical University, Zhanjiang 524002, China; 4 Department of Hospital Infection Management of Nanfang Hospital, Southern Medical University, Guangzhou 510515, China

**Keywords:** Atractylenolide I, sirtuin-6, hepatic steatosis, NACHT, LRR and PYD domains-containing protein 3, inflammasome

## Abstract

Accumulating evidence has highlighted that sirtuin-6 (SIRT6) plays an important role in hepatic gluconeogenesis and lipogenesis. We aim to investigate the underlying mechanisms and pharmacological interventions of SIRT6 on hepatic steatosis treatment. Herein, our results showed that atractylenolide I (ATL I) activated the deacetylase activity of SIRT6 to promote peroxisome proliferator-activated receptor alpha (PPARα) transcription and translation, while suppressing nuclear factor NF-kappa-B (NFκB)-induced NACHT, LRR, and PYD domains containing protein 3 (NLRP3) inflammasome formation. Together, these decreased the infiltration of F4/80 and CD11B positive macrophages, accompanied by decreased mRNA expression and serum levels of tumor necrosis factor alpha (TNF-α), interleukin-6 (IL6), and interleukin-1 beta (IL1β). Additionally, these changes decreased sterol regulatory element-binding protein-1c (SREBP-1c) expression, while restoring carnitine *O*-palmitoyltransferase 1a (Cpt1a) expression, to decrease the size of adipocytes and adipose deposition, which, in turn, reversed high-fat diet (HFD)-induced liver weight and body weight accumulation in C57 mice. SIRT6 knockout or hepatic SIRT6 knockout in C57 mice largely abolished the effect of ATL I on ameliorating hepatic steatosis. Taken together, our results suggest that ATL I acts as a promising compound that activates SIRT6/PPARα signaling and attenuates the NLRP3 inflammasome to ameliorate hepatic inflammation and steatosis.

## 1. Introduction

Hepatic steatosis is the initial stage of metabolic-associated fatty liver disease (MAFLD), and also presents as one of the most general hallmarks of chronic metabolic disease worldwide [1,2]. Hepatic steatosis is graded by the intrahepatic fat content, and is characterized as having an intrahepatic triglyceride (TG) level no less than 5% of liver weight, or by having 5% of hepatocytes contain lipid vacuoles without any other secondary causes [1,3]. Hepatic steatosis may be a benign and hepatoprotective state, unless hepatic adipose deposition is further accumulated and overloaded to transform into a pathological condition in order to trigger MAFLD [4,5,6,7].

Hepatic TG precursors and hepatic free fatty acids (FFAs) can be derived from food, lipolysis, or de novo lipogenesis, but hepatocytes usually do not reserve TG under physiologic conditions [7,8,9]. To a certain extent, accumulating adipose deposition in hepatocytes accounts for chronic disease progression, including MAFLD and cardiovascular diseases [10,11,12,13]. Mechanistically, protein phosphatase 2A catalytic alpha subunit (PP2A-Cα) and 5′-AMP-activated protein kinase (AMPK) are inactivated, while alpha serine/threonine-protein kinase (AKT) is activated, during hepatic steatosis progression [14,15]. Hepatic PPARα knockout significantly decreases peroxisome formation and lowers plasma β-hydroxybutyrate levels in order to elevate the proinflammatory M1 macrophage population and to reduce lipid metabolism for the purpose of promoting hepatic inflammation and hyperlipidemia [16]. Krüppel-like Factor 16 directly bonded the promoter of PPARα to increase FFA oxidation, reduce lipid deposition, and decrease the reactive oxygen species (ROS) level to ameliorate insulin resistance and steatohepatitis [17]. Toll-like receptor (TLR)/NF-κB signaling promoted the formation of the NLRP3 inflammasome, and then resulted in IL-1β maturation and caspase-1 activation to promote the initiation and progression of MAFLD [18,19]. Myeloid-specific NLRP3 ablation impaired the NLRP3 inflammasome to inhibit pro-inflammatory cytokine release [20]. Hepatic SIRT6 plays a hepatoprotective role, and decreases SREBP-1c to suppress hepatic lipogenesis [21,22,23]. In addition, previous studies highlighted that sirtuin family members commonly inactivated the NLRP3 inflammasome [24,25]. However, the effect of SIRT6 on regulating the NLRP3 inflammasome in hepatic steatosis progression remains elusive.

Generally, hepatic steatosis management includes exercise, diet control, and drug intervention. Emerging natural compounds and their derivatives have exhibited promising therapeutic effects on conditions including, but not limited to, liver diseases [18,26,27]. Atractylenolide III activates the hepatic adiponectin receptor 1 (AdipoR1)/AMPK axis to ameliorate non-alcoholic fatty liver disease [28]. 4-Methylesculetin increases CPT-1A, PPAR-α, and nuclear factor erythroid 2-related factor 2 Nrf2, while decreased CD36, PPAR-γ, SREBP-1, and fatty acid synthase (FASN) reduce hepatic lipid accumulation [29]. Lactucin and lactucopicrin target trifunctional enzyme subunit alpha (HADHA), disintegrin, metalloproteinase domain-containing protein 17 (ADAM17), Sequestosome-1 (SQSTM1), and lysosomal acid glucosylceramidase (GBA) genes to promote fatty acid oxidation, and then ameliorate FFA-induced steatosis [30]. ATL I is extracted from Atractylodes macrocephala Koidz, and plays multiple roles in anti-inflammation, anti-oxidation, and anti-tumor activities [31,32]. Interestingly, ATL I targets the TLR4/MAPKs/NF-κB axis to attenuate acetaminophen-induced acute liver injury [33]. However, the effect of ATL I and its underlying mechanism for regulating hepatic steatosis remain largely elusive.

In the present study, we isolated mouse primary hepatocytes (MPHs) and used a high-fat diet (HFD)-induced disease model to explore the effect of ATL I on regulating hepatic steatosis. In addition, we used SIRT6 KO, and especially hepatic SIRT6 KO, mice to confirm this effect, and then investigated the underlying mechanisms.

## 2. Results

### 2.1. ATL I Treatment Alleviates Lipid Accumulation In Vitro

**Figure 1:**
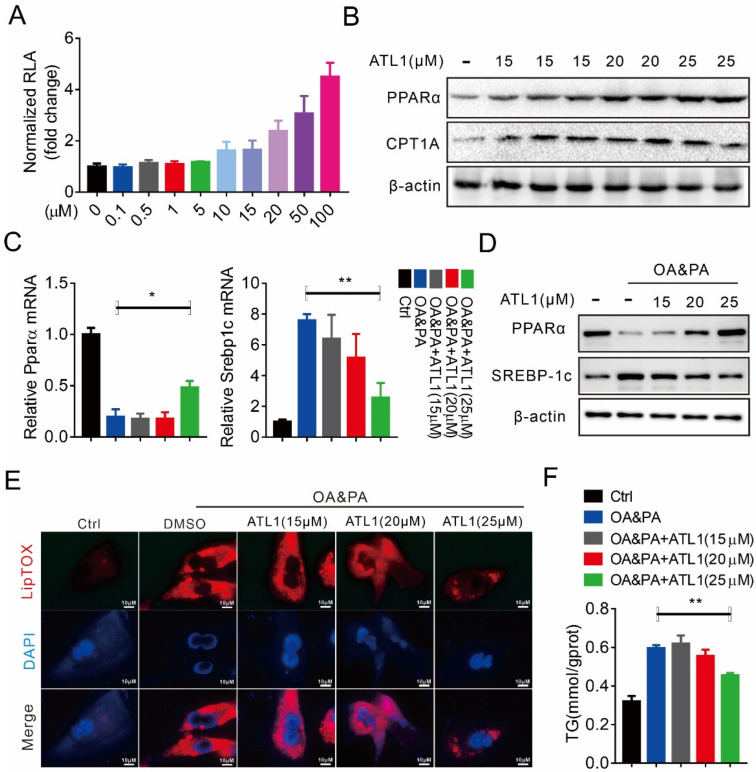
ATL I treatment alleviates lipid accumulation in vitro. (**A**) Luciferase reporter assays indicated that ATL I treatment effectively stimulated PPARα expression in HepG2 cells in a dosage-dependent manner. (**B**) ATL I treatment significantly increased the protein levels of PPARα and its target genes. (**C**,**D**) ATL I treatment effectively reversed the mRNA (**C**) and protein (**D**) levels of PPARα and SREBP-1c in OA&PA cultured MPHs. (**E**) LipidTOX assays indicated an effective reduction in lipid deposition in OA&PA treated MPHs after ATL I treatment. (**F**) ATL I treatment effectively decreased the TG contents in MPHs. Results are shown as mean ± SEM (n ≥ 3). * *p* < 0.05, ** *p* < 0.01 versus the OA&PA group.

We first investigated the effect of ATL I on hepatic steatosis, and our results showed that ATL I dose-dependently activated PPARα reporter activities (Figure 1A). PPARα and its downstream target CPT1A were consistently and dose-dependently increased after ATL I treatment (Figure 1B). PPARα mRNA was significantly decreased in mouse primary hepatocytes (MPHs) after oleate acid/palmitate acid (OA&PA) treatment. Lower doses of ATL I had no effect on regulating PPARα mRNA expression, but the highest dose of ATL I significantly and partially restored PPARα mRNA expression (Figure 1C). In contrast, Screbp-1c mRNA was significantly increased in mouse primary hepatocytes after OA&PA treatment, although lower doses of ATL I had no effect on regulating Screbp-1c mRNA expression. However, the highest dose of ATL I could significantly and largely reverse Screbp-1c mRNA expression (Figure 1C). OA&PA treatment also significantly decreased the PPARα protein level and increased the SCREBP-1c protein level in MPHs, but ATL I dose-dependently and largely reversed the OA&PA induced effects (Figure 1D). OA&PA treatment successfully induced steatosis in MPHs, and ATL I dose-dependently and significantly decreased OA&PA-induced steatosis (Figure 1E). In addition, ATL I also significantly reversed OA&PA-induced upregulation of TG levels in MPHs (Figure 1F).

### 2.2. ATL I Treatment Suppressed OA&PA-Induced NLRP3 Inflammasome Activation and the Inflammatory Response In Vitro

**Figure 2:**
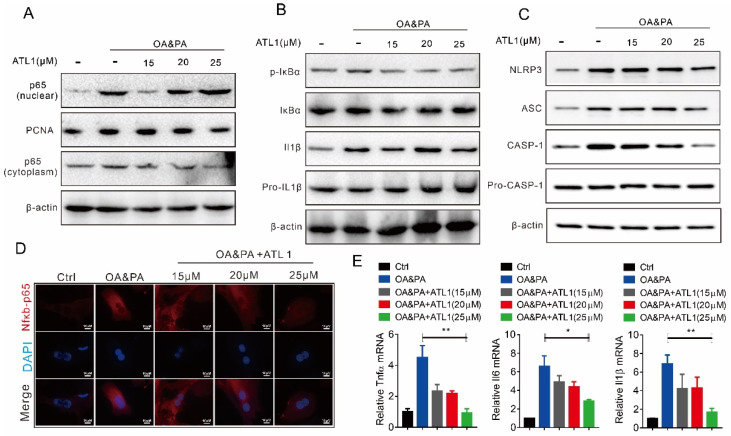
ATL I treatment suppressed OA&PA-induced NLRP3 inflammasome and inflammatory response in vitro. (**A**) Western blot indicated that ATL I treatment significantly decreased the OA&PA-induced p65 levels in the nuclei of MPHs. (**B**,**C**) ATL I treatment significantly decreased the OA&PA-induced expression of proteins related to NLRP3 inflammasome formation. (**D**) Immunofluorescence data indicated a decreased level of NfκB-p65 levels in OA&PA-treated MPHs after ATL I treatment. (**E**) ATL I treatment effectively reversed the mRNA levels of inflammatory genes in OA&PA cultured MPHs after ATL I treatment. Results are shown as mean ± SEM (n ≥ 3). * p < 0.05, ** p < 0.01 versus the OA&PA group.

We then further confirmed the effect of ATL I on regulating inflammation in MPHs. Our results showed that OA&PA treatment significantly increased the cytoplasmic and nuclear p65 protein levels, while ATL I dose-dependently suppressed p65 protein nuclear translocation (Figure 2A). There was no significant difference in NF-kappa-B inhibitor alpha (IκBα) or pro-IL1β protein levels with or without ATL I treatment, but ATL I treatment decreased OA&PA-induced IκBα phosphorylation levels and IL1β maturation (Figure 2B). There was also no significant difference in the pro-Caspase-1 (pro-CASP-1) protein level with or without ATL I treatment, but it decreased OA&PA-induced NLRP3, an apoptosis-associated speck-like protein containing a CARD (ASC), as well as CASP-1 activation (Figure 2C). Immunofluorescence experiments also showed that OA&PA treatment significantly increased the p65 cytoplasmic protein levels and promoted its nuclear translocation. This effect of OA&PA treatment was dose-dependently reversed by ATL 1 treatment (Figure 2D). OA&PA treatment significantly increased the mRNA expression of Tnfα, IL6, and IL1β, while ATL I dose-dependently reversed this effect of OA&PA treatment (Figure 2E).

### 2.3. ATL I Treatment Alleviates HFD-Induced Fatty Liver in Mice

**Figure 3:**
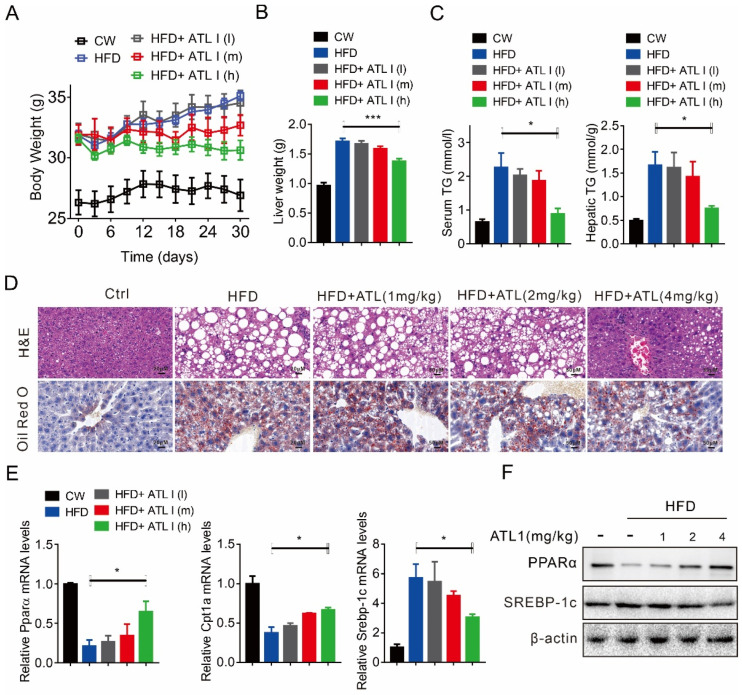
ATL I treatment alleviates HFD-induced fatty liver in mice. (**A**) ATL I treatment effectively decreased the body weight of HFD-induced obese mice in a dosage-dependent manner. (**B**) ATL I treatment significantly decreased the liver weight of HFD-induced obese mice. (**C**) ATL I treatment effectively alleviated HFD-induced elevation of TG contents in serum and liver of HFD-induced obese mice. (**D**) H&E and oil red O staining data indicated an improvement of fatty liver phenotype and lipid accumulation in HFD-induced obese mice after ATL I treatment. (**E**,**F**) ATL I treatment effectively attenuated the mRNA (**E**) and protein (**F**) levels of genes related to lipid metabolism in HFD-induced obese mice. Results are shown as mean ± SEM (n ≥ 4). * p < 0.05, *** p < 0.001 versus the HFD group.

We then evaluated the effect of ATL I on hepatic steatosis. ATL I dose-dependently decreased the body weight of HFD-fed mice (Figure 3A). In addition, ATL I dose-dependently decreased liver weight in HFD-fed mice, but there was a significant difference only in the highest dose treatment (Figure 3B). ATL I dose-dependently decreased serum and hepatic triglycerides in HFD-fed mice, but there was a significant difference only in the highest dose treatment (Figure 3C). ATL I dose-dependently decreased the size of adipocytes and adipose deposition in HFD-fed mice (Figure 3D). In addition, ATL I dose-dependently restored the mRNA expression of Pparα and Cpt1a, but dose-dependently decreased the mRNA expression of Srebp-1c in HFD-fed mice (Figure 3E). Consistently, ATL I dose-dependently restored PPARα protein levels, while it dose-dependently decreased SREBP-1c protein levels in HFD-fed mice (Figure 3F).

### 2.4. ATL I Treatment Suppressed NLRP3 Inflammasome and Inflammatory Response in HFD-Induced Obese Mice

**Figure 4:**
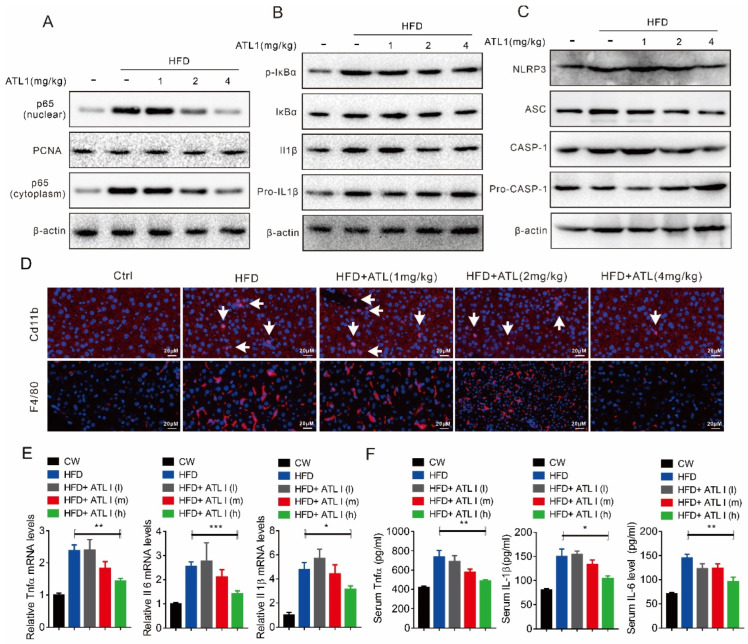
ATL I treatment suppressed NLRP3 inflammasome and inflammatory response in HFD-induced obese mice. (**A**) Western blot indicated that ATL I treatment significantly decreased the HFD-induced p65 levels in the nuclei of mouse livers. (**B**,**C**) ATL I treatment significantly decreased the HFD-induced expression of proteins related to NLRP3 inflammasome formation in mouse livers. (**D**) Immunofluorescence data indicated a decreased level of Cd11b and F4/80 positive cells in HFD-induced obese mice after ATL I treatment. (**E**) ATL I treatment effectively reversed the mRNA levels of inflammatory genes in HFD-induced obese mice after ATL I treatment. (**F**) ELISA data showed an obviously decreased level of serum inflammatory cytokines levels in HFD-induced obese mice after ATL I treatment. Results are shown as mean ± SEM (n ≥ 4). * p < 0.05, ** p < 0.01, *** p < 0.001 versus the HFD group.

We also investigated the effect of ATL I on the inflammatory response in HFD-fed mice. In line with the in vitro study, our results showed that HFD feeding significantly increased the cytoplasmic and nuclear p65 protein levels, while ATL I dose-dependently suppressed p65 protein nuclear translocation (Figure 4A). There was no significant difference in the IκBα protein levels with or without ATL I treatment, but it decreased the HFD-induced IκBα phosphorylation level (Figure 4B). HFD feeding led to an increase in pro-IL1β protein levels and promoted their maturation, while ATL I dose-dependently reversed this effect of HFD feeding (Figure 4B). There was also no significant difference in pro-CASP-1 protein levels with or without ATL I treatment, but it decreased HFD-induced NLRP3, ASC, and CASP-1 activation (Figure 4C). HFD feeding significantly increased the infiltration of F4/80 and CD11B positive macrophages and increased the mRNA expression and serum levels of Tnfα, IL6, and IL1β, while ATL I dose-dependently reversed this effect of HFD feeding (Figure 4D,F).

### 2.5. Hepatic SIRT6 Mediates the Protective Effects of ATL I Treatment against Lipid Disorder

**Figure 5:**
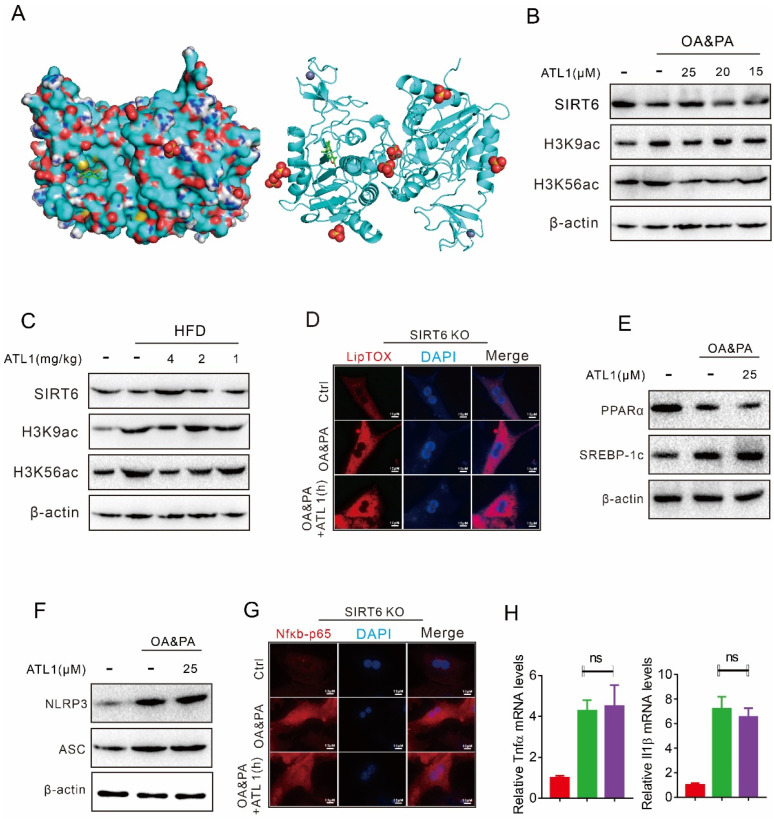
Hepatic SIRT6 mediates the protective effects of ATL I treatment against lipid disorder and inflammation response. (**A**) Molecular docking analysis showed a perfect binding between SIRT6 and ATL I. (**B**) ATL I treatment significantly increased the expression of SIRT6, H3K9ac, and H3K56ac in MPHs. (**C**) ATL I treatment significantly increased the expression of SIRT6, H3K9ac, and H3K56ac in mouse livers. (**D**) LipidTOX assays indicated that ATL I treatment failed to alter the lipid deposition in SIRT6-deficient MPHs. (**E**) ATL I treatment failed to alter the expression of PPARα and SREBP-1c in SIRT6-deficient MPHs. (**F**) ATL I treatment failed to decrease NLRP3 and ASC protein levels in SIRT6-deficient MPHs. (**G**) ATL I treatment failed to decrease the nuclear NfκB-p65 levels in SIRT6-deficient MPHs. (**H**) ATL I treatment failed to decrease the inflammatory genes in SIRT6-deficient MPHs. Results are shown as mean ± SEM (n = 4).

We then performed a molecular docking analysis and found that there was a perfect binding between SIRT6 and ATL I (Figure 5A). OA&PA treatment or HFD feeding decreased the SIRT6 protein level, while increasing the H3K9ac and H3K56ac protein levels in vitro (Figure 5B). Interestingly, ATL I treatment dose-dependently restored SIRT6 protein levels and decreased H3K9ac and H3K56ac protein levels in vivo (Figure 5C). SIRT6 knockout (KO) in mice attenuated the effect of ATL I on suppressing OA&PA-induced steatosis in the MPHs of SIRT6 KO mice (Figure 5D). SIRT6 KO in mice also attenuated the effect of ATL I on restoring OA&PA-decreased PPARα protein levels and suppressing OA&PA-induced SREBP-1c protein levels (Figure 5E). SIRT 6 deficiency also attenuated the effect of ATL1 on decreasing NLRP3 and ASC protein levels (Figure 5F). SIRT6 KO, in mice, attenuated the effect of ATL I on suppressing OA&PA-induced NFκB-p65 nuclear translocation (Figure 5G). In addition, SIRT6 KO, in mice, attenuated the effect of ATL I on suppressing OA&PA-induced mRNA expression of Tnfα and IL1β (Figure 5H).

### 2.6. ATL I Treatment Failed to Alter HFD-Induced Fatty Liver in Hepatic SIRT6 Knockout Mice

**Figure 6:**
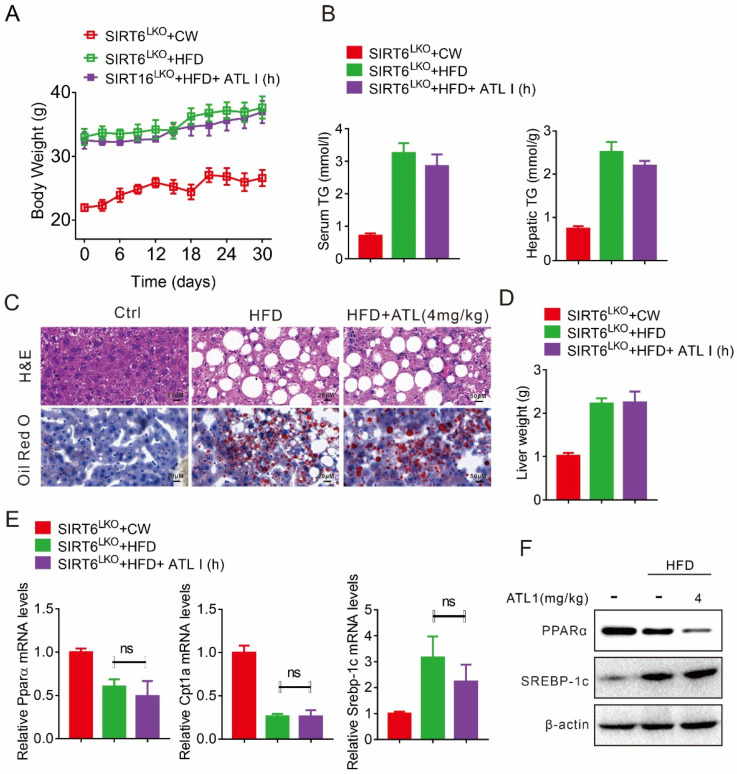
ATL I treatment failed to alter HFD-induced fatty liver in hepatic SIRT6 knockout mice. (**A**) ATL I treatment did not change the body weight of hepatic SIRT6 knockout mice fed an HFD. (**B**) ATL I treatment failed to change the TG levels in the serum and livers of hepatic SIRT6 knockout mice fed an HFD. (**C**) H&E and oil red O staining data indicated that ATL I treatment failed to change the fatty liver phenotype and lipid accumulation in hepatic SIRT6 knockout mice fed an HFD. (**D**) ATL I treatment did not change the liver weight of hepatic SIRT6 knockout mice fed an HFD. (**E**,**F**) ATL I treatment failed to alter the mRNA (**E**) and protein (**F**) levels of genes related to lipid metabolism in hepatic SIRT6 knockout mice fed an HFD. Results are shown as mean ± SEM (n ≥ 4).

We then constructed hepatic SIRT6 knockout mice to evaluate its effect on the response to ATL I treatment. Our results showed that ATL I could not decrease the body weight of hepatic SIRT6 knockout (KO) mice fed a HFD (Figure 6A). Hepatic SIRT6 KO, in mice, attenuated the effect of ATL I on suppressing HFD-induced serum and hepatic triglycerides (Figure 6B). Hepatic SIRT6 KO in mice attenuated the effect of ATL I on suppressing HFD-induced adipocyte expansion, hepatic adipose deposition, and liver weight accumulation (Figure 6C,D). ATL I neither restored the mRNA expression of Pparα and Cpt1a in hepatic SIRT6 KO mice under HFD feeding conditions, nor reversed HFD-induced mRNA expression of Srebp-1c (Figure 6E). In addition, hepatic SIRT6 KO in mice attenuated the effect of ATL I on restoring HFD-decreased PPARα protein levels and suppressing HFD-induced SREBP-1c protein levels (Figure 6F).

### 2.7. Hepatic SIRT6 Deletion Diminished the Protective Effects of ATL I

**Figure 7:**
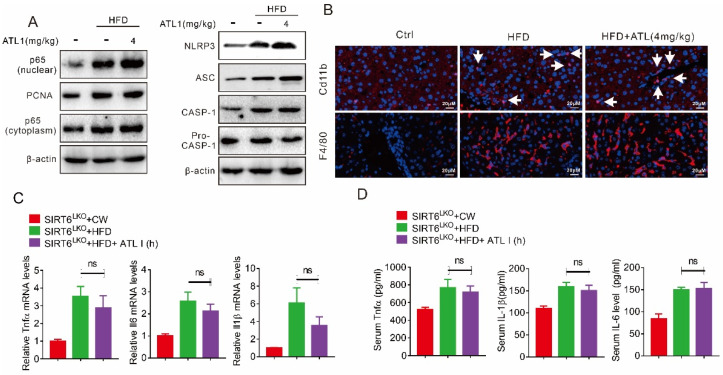
Hepatic SIRT6 deletion diminished the protective effects of ATL I treatment on the NLRP3 inflammasome and inflammatory response in HFD-induced mice. (**A**) Western blot indicated that ATL I treatment failed to decrease the HFD-induced p65 levels in nuclear and NLRP3 inflammasome related proteins in the livers of hepatic SIRT6 knockout mice. (**B**) Immunofluorescence data indicated that an ATL I-induced decrease in Cd11b and F4/80 positive cells in HFD-induced obese mice was abolished due to hepatic SIRT6 knockout. (**C**) ATL I treatment failed to reverse the mRNA levels of inflammatory genes in hepatic SIRT6 knockout mice fed an HFD. (**D**) ELISA data showed no change in serum inflammatory cytokine levels in hepatic SIRT6 knockout mice fed an HFD after ATL I treatment. Results are shown as mean ± SEM (n ≥ 4).

Our results further showed that ATL I could not inhibit HFD-induced p65 nuclear translocation in SIRT6 KO mice, and could not reverse the effect on either HFD-induced NLRP3 and ASC protein expression or CASP-1 maturation (Figure 7A). Hepatic SIRT6 KO in mice attenuated the effect of ATL I on suppressing HFD-induced macrophage infiltration (Figure 7B). In addition, ATL I could not suppress HFD-induced mRNA expression or serum levels of Tnfα, IL6, and IL1β in the SIRT6 KO mice (Figure 7C,D).

## 3. Discussion

**Figure 8:**
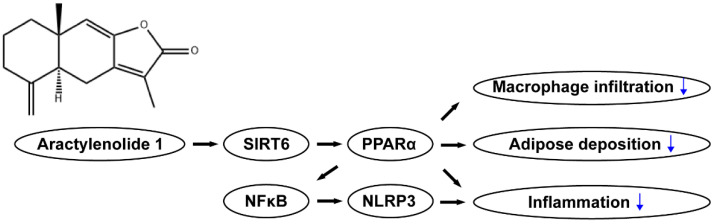
Schematic diagram demonstrating how ATL I alleviates hepatic steatosis.

In the present study, we performed a molecular docking analysis and found that SIRT6, as the ATL I targeted effector, increased deacetylation activity to activate PPARα and also increased its target genes to accelerate fatty acid oxidation, accompanied by attenuating NFκB-mediated NLRP3 inflammasome formation to inhibit hepatic lipid accumulation-induced hepatic inflammation and steatosis (Figure 8).

ATL-I, -II, and -III are extracted from Atractylodes macrocephala Koidz, a traditional Chinese herb that has been widely used for its antioxidant, anti-inflammatory, neuroprotective, and anticancer properties [34]. Otherwise, ATL III activates the hepatic AdipoR1/AMPK/SIRT1 axis to ameliorate HFD- or FFA-induced MAFLD [28]. Cytochrome P450 can oxidize ATL II to ATL III, while ATL III can be dehydrated to ATL I [35]. ATL I remarkably inhibited the bacille Calmette-Guérin (BCG) vaccine and lipopolysaccharides (LPS), ameliorating immunological liver injury, or acetaminophen-induced acute liver injury [33,36]. Thus, these previous studies allowed us to investigate the effect of ATL I on hepatic fat metabolism and MAFLD. In the present study, our results confirmed that ATL I exhibited a promising effect that inhibited inflammatory cell infiltration and, subsequently, inflammatory factor release. It also decreased hepatic adipose deposition to reverse HFD-induced liver weight and body weight accumulation, which, together, would be beneficial for hepatic steatosis treatment.

In the present study, we first explored the underlying mechanism by which ATL I regulates hepatic inflammation. Interestingly, we performed the molecular docking analysis to find an ATL I binding protein, SIRT6. Emerging evidence has suggested that ATL I/III targets SIRT1 to play important roles in energy metabolism, anti-inflammation, and autophagy activation [37,38,39]. Otherwise, both ATL I and SIRT6 showed a neuroprotective activity. ATL I inactivated the NF-κB and mitogen-activated protein kinase (MAPK) signaling pathways to decrease the expression of TNF-α, IL-6, and IL-1β. It also induced nitric oxide synthase (iNOS) and cytochrome c oxidase subunit 2 (COX-2) to suppress neuroinflammation [40]. ATL I also decreased the ratio of Bax/Bcl-2 and inhibited caspase activation to attenuate oxidative stress injury [41]. SIRT6 inhibits thioredoxin-interacting protein (TXNIP) to silence microglia-induced inflammation and promote potentiated angiogenesis to ameliorate cerebral ischemic damage [42]. SIRT6 activates the histone-lysine N-methyltransferase (EZH2)/forkhead box protein C1 (FOXC1) axis to ameliorate neuroinflammation and cerebral ischemic damage [43]. These studies caused us to speculate that ATL I targets SIRT6 to suppress hepatic inflammation. Indeed, our results further showed that SIRT6 activation by ATL I attenuated NFκB-mediated NLRP3 inflammasome formation. According to earlier studies, NLRP3 activation in macrophages plays an important role in NASH [44,45]. Our results have shown that the Cd11b and F4/80 positive cells were decreased in HFD-induced obese mice after ATL I treatment. Thus, macrophages were also one of the target cell types of ATL I. However, we have not yet examined the NLRP3 activation signaling in macrophages. In a subsequent study, we would prefer to investigate the protective effect of ATL I on hepatocytes. However, we agree with the points on the importance of NLRP3 activation in macrophages involved in the development of NAFLD and liver fibrosis, and we will continue to perform related studies in the future.

A previous study showed that SIRT6 interacted with spectrin beta chain, non-erythrocytic 1 (SPTBN1) to crosstalk with TGF-β signaling, and contributed to fatty liver disease [46]. PPARs are key regulators of lipid metabolism [17,47,48,49,50]. SIRT6 also activated PPARα to repress SREBP-dependent cholesterol and triglyceride synthesis to maintain proper hepatic fat content [51]. In the present study, our results showed that ATL I dose-dependently elevated PPARα and its target genes to accelerate fatty acid oxidation. SIRT6 KO, and especially hepatic SIRT6 KO, abolished these effects of ATL and failed to attenuate hepatic steatosis in mice.

Actually, most of our results only showed significant efficacy in the highest dose (4 mg/kg) ATL I group. This might raise the concern that a higher dose of the drug would be accompanied by more severe adverse effects. Our in vivo study showed that all treatment groups exhibited a good tolerance during the experiments. The highest dose of ATL I we used here provided a protective and promising role in body weight control and decreased hepatic adipose deposition. In addition, other studies have used 60 or 120 mg/kg ATL I to ameliorate acetaminophen-induced acute liver injury [33]. Another study used 10 to 40 mg/kg ATL I to inhibit aldosterone synthesis and improve hyperaldosteronism [52]. Taking these together, we believe that the dose of ATL I we used in the present study was safe. This might also incline us to test higher doses (over 4 mg/kg) of ATL I in future studies. Accumulating evidence has indicated that ATL I has a promising effect on cancer treatment, at least including colon cancer, cervical cancer, and breast cancer [53,54,55,56,57]. Thus, this also inclines us to explore the effect of ATL 1 on treating the later stages of metabolic-associated fatty liver disease, such as liver fibrosis, and even liver cancer.

## 4. Materials and Methods

### 4.1. Animal Treatment

Eight-week-old male C57BL/6J mice were purchased from Guangdong Medical Laboratory Animal Center. Hepatic SIRT6 knockout mice were established as previously reported by crossing Alb-Cre mice with SIRT6fl/fl mice. All mice were housed with a 12 h light/dark photoperiod and with unrestricted water and food. To establish diet-induced obesity models, mice were fed a high-fat diet (D12492, Research Diets, New Brunswick, NJ, USA) for 8 weeks before ATL I (CSN16457, CSNpharm, Shanghai, China) (high dose (h): 4 mg/kg, ATL I medium dose (m): 2 mg/kg, and ATL I low dose (l): 1 mg/kg) treatment for another four weeks. Dimethyl sulfoxide (DMSO) (ST2335, Beyotime, Shanghai, China) was set as vehicle, and saline was set as negative control [52]. All animal care and experimental studies were approved by and in accordance with the guidelines of the Animal Ethics Committee of Guangdong Medical University. At the endpoint of the experiments, the mice were fasted for 6 h, and then the liver tissues and plasma were collected for subsequent experiments.

### 4.2. Isolation of Mouse Primary Hepatocytes (MPHs)

The MPHs were extracted by an in situ two-step perfusion method, as previously reported [54]. In brief, mice were anesthetized with 10% chloral hydrate, and the limbs were fixed. Then, the abdomen was disinfected with 75% ethanol and the abdominal cavity was opened to fully expose the portal vein. AKRB slow flush solution at 37 °C was aspirated into a 20 mL syringe, the needle was entered into the hepatic portal vein, and the solution was perfused at a rate of 4 mL-min-1 until the liver turned grey-yellow; then, the solution was replaced with a 0.05% collagenase type IV (C4-22-1G, Sigma, St. Louis, MO, USA) solution and perfused in the same way. The liver was removed, placed in a sterile Petri dish, mashed and ground to allow for complete cell release, and then filtered. The filtered cytosol was aspirated into a centrifuge tube, and RPMI 1640 medium (G4530, Servicebio, Wuhan, China), containing 10% fetal bovine serum (G8001, Servicebio, Wuhan, China) (FBS) and 1% penicillin-streptomycin solution (C0222, Beyotime, Shanghai, China), was added. The supernatant was discarded by centrifugation and the pellet was incubated in a 5% CO_2_, 37 °C incubator. When the cells grew to occupy 80–90% of the dish, passages or experiments were performed.

### 4.3. Molecular Docking

Molecular docking was carried out as previous reported [56]. In brief, the ATL I formula was obtained from the ChemSpider Database (ChemSpider ID4478915). The energy structure of the molecule can be partially optimized by means of a molecular mechanical procedure (Minimize) to obtain a stable conformation with low energy. The 3D protein crystal structure of SIRT6 was acquired from the Protein DataBank of RCSB (http://www.rcsb.org, accessed on 6 June 2022). Using Surflex, the high precision pattern on the Dock module analyzed ATL 1 and SIRT6 flexible docking target proteins, according to the electrostatic interaction, hydrogen bonding interaction, van der Waals interactions, hydrophobic interaction simulation, and evaluation of small molecules to the protein binding patterns and interactions. The docking results were analyzed using Discovery Studio 2016 software.

### 4.4. Western Blotting

The cells were incubated with OA&PA (O1008 and P5585, Sigma, St. Louis, MO, USA) for 24 h after ATL I dosing for 24 h. The cells were divided into a normal group, a model group, and a model dosing group. The cells were collected and subjected to protein extraction, electrophoretic separation, membrane transfer, and closure at room temperature for 1 h. The primary antibodies were incubated at 4 °C overnight, followed by secondary antibody incubation for 1 h, development under a gel imager, and imaging photography, according to the Western blot procedure. Protein expression levels were analyzed.

### 4.5. Luciferase Reporter Assays

Liver primary cells were seeded in 24-well plates and maintained in 1640 medium containing 1% penicillin-streptomycin solution and 10% FBS. After cell apposition, cells were co-transfected using Lipofectamine™ 2000 (11668019, Invitrogen, Carlsbad, CA, USA), with each indicated luciferase reporter gene and expression plasmid. Co-infection was balanced with the same total amount of DNA by using the pcDNA3.1 vector. The transfection efficiency was adjusted using the Alenira luciferase expression vector pCMV-RL-TK as an i-type control. Luciferase activity was detected at 48 h post-transfection.

### 4.6. Real Time PCR

Total RNA was extracted from primary mouse hepatocytes using the Trizol reagent (15596026, Thermo Fisher, Waltham, MA, USA), and then assayed for RNA concentration (A260/A280 = 1.8 to 2.0). Then, the RNA was reverse transcribed using Premix Ex Taq™ II (RR820B, Takara, Dalian, China). Finally, PCRs were performed, and lysis curve analysis was performed after amplification to determine whether the amplification was specific. Data were normalized and analyzed with the internal reference β-actin. CT values were calculated using 2-ΔΔCT to calculate the mRNA transcript levels for each group. The primer design and synthesis were performed by Beijing DynaBio.

### 4.7. Immunofluorescence

The MPH was incubated with NF-κB (A19653, Abclonal, Wuhan, China), and liver tissue was incubated with primary antibodies against F4/80 (A18637, Abclonal, Wuhan, China) and CD11b (A1581, Abclonal, Wuhan, China) overnight at 4 °C. After washing three times with PBS, the sections were incubated with secondary antibody (AS039 and AS008, Abclonal, Wuhan, China) for 40 min at room temperature. After washing five times with phosphate-buffered saline (PBS) (C0221A, Beyotime, Shanghai, China), images were observed with a fluorescence microscope (ECLIPSE Ti2-U, Nikon, Tokyo, Japan). 

### 4.8. TG Content Test

The cells were cultured in 6-well plates and divided into 3 groups: the normal group, model group, and model plus group. After 24 h of OA&PA modeling, ATL I was added for 24 h. Then, 100 μL of cell lysate was added to each of the 6-well plates, and the cells were transferred to 1.5 mL EP tubes with a cell scraper and rotated repeatedly to fully lyse the cells. The cells were allowed to stand for 10 min at room temperature, and then centrifuged at a radius of 8 cm for 2 min at 500 r-min^−1^. The supernatant was collected, and the TG content of the cells was measured using an automatic biochemical analyzer.

### 4.9. Lipid TOX Assays

The cells were incubated in six-well plates and treated with OA&PA for 24 h. After 24 h of continued drug intervention, the medium was aspirated and discarded, washed three times with PBS, and diluted HCS Lipid TOXTM Red (1:1000) (H34477, Thermo Fisher, Waltham, MA, USA) was added. The cells were incubated at 37 °C for 30 min, the drug-containing culture medium was aspirated and discarded, and the slices were sealed with a sealer containing DAPI (C1002, Beyotime, Shanghai, China) and then observed under a fluorescence microscope at 100×.

### 4.10. Nuclear Protein Extraction

A nuclear protein extraction experiment was carried out as previous reported [57]. In brief, the cells were washed with PBS at the end of the treatment, and the cells were scraped off with a cell scraper, leaving the cell precipitate. Cell plasma proteins and nuclear proteins were separated with a Cell Nucleoprotein and Cell Plasma Protein Extraction Kit (P0028, Beyotime, Shanghai, China). The proteins were denatured and subjected to conventional electrophoretic electrotransfer, then incubated with NF-kB p65/RelA rabbit mAb (A19653, Abclonal, Wuhan, China) overnight for 4 h, and with a secondary antibody, IgG H&L (ab6702, Abcam, Cambridge, UK), for 1 h. They were then developed under a gel imager and imaged according to the Western blot procedure. Protein levels were analyzed.

### 4.11. HE and Oil Red O Staining

H&E and oil red O staining was performed as previously reported [56]. Briefly, for H&E staining, indicated liver tissues were fixed in 10% neutral-buffered formalin, embedded in paraffin, and cut into 7-µm sections. For oil Red O staining, liver tissue was frozen in liquid nitrogen and cut into 10-µm sections. Sections were stained and analyzed at 20× magnification using a microscope.

### 4.12. Statistical Analysis

The data of the present study are shown by the mean ± standard error (SE). Student’s *t*-test was used to analyze the differences in continuous variables between the two groups. Tukey’s multiple comparison test of ANOVA was performed to assess the significance of all data. Statistical significance was accepted when *p* < 0.05.

## 5. Conclusions

In the present study, our results extended a promising application of ATL I in hepatic steatosis treatment. We also provided an alternative mechanism by which ATL I binds with SIRT6 to activate PPARα and its target genes and suppresses inflammatory factor release by attenuating NFκB-mediated NLRP3 inflammasome formation, which, together, attenuate hepatic inflammation and steatosis.

## Data Availability

Data is contained within the article.
